# A Case Report: 19-Year-Old Hispanic Young Woman with Early-Stage Breast Cancer and a Germline Pathogenic Variant

**DOI:** 10.18103/mra.v13i2.6293

**Published:** 2025-02-08

**Authors:** Tarsha Jones, Alice Zhang, Katherine Freeman

**Affiliations:** 1 Florida Atlantic University, Christine E. Lynn College of Nursing, Boca Raton, FL 33431; 2 Florida Atlantic University Summer Institute in Biostatistics and Data Science Program Alumni; Data Science BA Candidate, Wellesley College, MA 02481; 3 Florida Atlantic University, Schmidt College of Medicine, Boca Raton, FL 33431

## Abstract

**Background:**

Breast cancer (BC) is the most common cancer diagnosed in women globally and the leading cause of cancer-related deaths among younger women diagnosed between the ages of 20–49 years in the United States (US). Although the median age of BC diagnosis is 62 years overall, recent data show that early-onset BC is on the rise in young people. Black and Hispanic women are disproportionately affected by BC, often diagnosed at a younger age, and BC is the leading cause of cancer-related deaths for both groups of women. The aim of this report is to present the unique case of a young adult Hispanic woman diagnosed with early-stage BC. Younger women with BC face unique biological and psychosocial challenges compared to older post-menopausal women. Additionally, young breast cancer survivors (YBCS) are at an increased risk of BC recurrence. Therefore, there is a critical need to develop interventions that are tailored to the cultural and age-specific needs of racial and ethnic minority women to promote cancer risk-reduction and to improve health outcomes.

**Method: Case Report:**

We present the case of a 19-year-old Hispanic female with no family history of breast cancer (BC), who was diagnosed with ductal carcinoma in situ (DCIS). Initially diagnosed in 2018, she received treatment at a comprehensive cancer center. At the time of diagnosis, she completed multigene panel testing to identify hereditary cancer risk. The testing revealed a pathogenic variant in the *PTEN* gene. The patient underwent a bilateral mastectomy as part of her treatment plan due to her high-risk status. Five years post-diagnosis, in 2023 she joined our NIH-funded research study focused on investigating the experiences and needs of young breast cancer survivors (YBCS) from diverse racial and ethnic backgrounds.

**Conclusion:**

Women are being diagnosed with BC at increasingly younger ages. This case underscores the need to increase awareness about risk factors among diverse younger women and highlights the critical role of genetic testing in identifying hereditary breast and ovarian cancer (HBOC) syndrome and using that knowledge for informed decision-making. This is especially important for racial and ethnic minority women who suffer disproportionately from BC outcomes. Furthermore, it emphasizes the importance of a holistic approach to care, grounded in caring science, which prioritizes the well-being of the whole person. Nurses, in collaboration with other healthcare providers, are in a unique position to positively impact the lives of young breast cancer patients, survivors, and those at high-risk for developing the disease. By advocating for early detection, tailored age-appropriate interventions, and comprehensive support, nurses can significantly improve health outcomes and empower young women to live longer, healthier lives. Future research should investigate the complex interaction between biological, psychological, social, and environmental factors in order to reduce cancer health disparities and improve health for all.

## Introduction

Breast cancer (BC) is the leading cause of cancer-related deaths among women aged 20–49 years in the United States (US).^1–3^ Many people may perceive BC as a disease affecting older post-menopausal women in their 50’s or 60’s, but young women *can* and *do* get breast cancer.^4,5^ An estimated 16% of new invasive BC cases and 17% of non-invasive, early-stage, ductal carcinoma in situ (DCIS) cases are diagnosed in women younger than 50, although the median age at diagnosis is 62.^1,6^ Recent data show that early-onset cancers are on the rise among young people,^2,7^ highlighting a significant and urgent public health issue that demands greater attention.

Previous studies have found that breast cancer (BC) occurring in younger women has less favorable outcomes.^2,8,9^ Younger women face unique physical and mental health challenges that differ from those of older women, particularly due to their critical life stage and the diagnosis occurring during their reproductive, child-bearing years. Alarmingly, racial and ethnic disparities persist, with BC being the leading cause of cancer-related deaths for young Black and Hispanic women.^2,10,11^

When BC occurs at a younger age, it tends to be more biologically aggressive, with a higher likelihood of germline pathogenic variants *(i.e., BRCA1/2* genes), and younger women face greater odds of recurrence or developing a second breast primary compared to older women, which can lead to pre-mature deaths.^4–8^ Pathogenic variants or mutations in the *BRCA1* and *BRCA2* (breast cancer genes) significantly increases a woman’s lifetime risk of developing breast or ovarian cancer.^12^ While the average woman has a 13% risk of developing BC, this risk increases exponentially to 55%−70% for women with *BRCA1* variant carriers and 45–70% for those with a *BRCA2* variant. Furthermore, about 25%−40% of BC survivors with inherited harmful *BRCA1/2* mutations will develop contralateral BC within 20 years of their first diagnosis, compared to 8% of the general population. In addition to *BRCA1/2*, several other non-*BRCA* genes are associated with hereditary breast and ovarian cancer (HBOC) syndrome and have been identified, including highly penetrant genes such as *PTEN, TP53, CDH1*, and *STK11* and moderate penetrant genes like *ATM, CHEK2, PALB2, BRIP1, BARD1, RAD51C*, and *RAD51D*.^13–15^ The significantly elevated risk of developing BC for women with pathogenic variants underscores the importance of completing multigene panel testing in young BC patients.^4,15–17^

Beyond the medical implications, younger women often face profound psychosocial challenges, including anxiety, depression, fear of recurrence, body image concerns post-surgery, fertility challenges, dating and relationship issues, parenting concerns, and financial strain.^8,18,19^ The aim of this report is to highlight a unique case of a young woman with BC who has a pathogenic variant in the *PTEN* gene, underscoring the critical need to increase awareness of early-onset breast cancer, its associated genetic risk, and the unique challenges of being young and diagnosed with breast cancer.

## Case Report

Here, we present the case of a 19-year-old Hispanic female diagnosed with breast cancer, exemplifying many of these challenges. She reported experiencing initial symptoms, such as nipple discharge from her left breast and feeling a lump, which prompted her to visit her primary care provider (PCP) for evaluation. She reported that while she did not have cancer on her radar she knew to get checked and stated the following, “*as soon as my breast began to leak and the lump was beginning to be palpable, that’s when I knew I should probably get it checked out.”* Initially diagnosed in 2018, she underwent treatment at a comprehensive cancer center. Five years post-diagnosis, in 2023, she joined our NIH-funded research study focused on young breast cancer survivors (YBCS) from diverse racial and ethnic backgrounds. At the time of presentation, the patient was a student, unemployed, and covered by Medicaid insurance, highlighting the intersection of medical, social, and financial challenges experienced by young breast cancer patients.

Her clinical evaluation included a breast ultrasound and diagnostic mammogram, which revealed suspicious findings. She had a core needle biopsy in 2018, which confirmed the diagnosis of stage 0, early-stage breast cancer or DCIS. Given her young age at diagnosis, she completed germline genetic testing to identify genetic cancer risk. The patient reported no family history of BC or other cancers. Her medical history was notable for a previous surgery to remove a tumor on her spine in 2014. She did not smoke or drink alcohol. Multigene panel testing at the time of her initial BC diagnosis, confirmed a pathogenic variant or mutation in the *PTEN* gene. The patient reported that her initial treatment plan did not include adjuvant chemotherapy or radiation therapy. *PTEN*, phosphatase and tensin homolog, is a highly penetrant tumor suppressor gene, and a pathogenic variant in this gene is associated with up to 65%−85% increased risk of BC.^13,20^ Pathogenic variants in PTEN gene are commonly found in various tumors, including those of the brain, lungs, breast, liver, pancreas, and prostate.^20^ Additionally, *PTEN* mutations are associated with Cowden syndrome, a genetic condition characterized by multiple benign tumor-like growths (hamartomas) and an elevated risk of certain cancers.

By the time she joined our study, she was a breast cancer survivor; however, she reported living with a chronic liver condition and was awaiting a liver transplant. The patient shared several psychosocial challenges she faced as a result of her breast cancer diagnosis and treatment. On a global health scale, she rated her overall health as “poor” and rated her post-cancer quality of life as “fair”. The patient reported experiencing severe fatigue but described her mood as “good,” noting that she has a therapist who helps her to manage her mental and emotional health needs. She shared the following:

*“The loss of my breasts caused me to feel depressed. It feels like a piece of me was taken away. I have been seeing a therapist twice per week on zoom since my cancer diagnosis. It’s been very helpful for me to manage my emotions better and help me with personal relationships. The cancer was the roughest on me mentally. None of my other surgeries were as bad as the cancer one. At least with other surgeries I can still have a bit of normalcy despite the scars.”* She also stated:

“There’s not much to say, I wish there was more access for young women who go through it. I was lucky enough I had my partner’s support to guide me to therapy to come to acceptance with what happened to me. But in my health front, I have never been able to connect with someone my age.”

## Discussion

This case report is important for several reasons. Alarmingly, recent data shows that early-onset cancers are on the rise among young adults, highlighting the need for greater awareness of this issue due to the unique challenges faced by this population. A recent *BBC* science report highlights the rising cases of breast cancer, colorectal, and other cancers in young adults in their 20’s and 30’s.^2,7,21,22^ Notably, the rising cases of BC in young women present unique challenges and is an urgent public health issue.^2,7,21–25^ The Centers for Disease Control and Prevention’s (CDC) *Morbidity and Mortality Weekly Report* (MMWR) found that among women aged 20–39, the incidence of BC increased by 0.7% per year from 2010–2018, while among women aged 40–49, incidence increased by 0.4% per year between 2002 and 2018.^22^

Additionally, a recent study published in *JAMA* analyzed 217,815 cases of primary invasive BC among women aged 20–49, including participants from diverse racial and ethnic backgrounds: American Indian/Alaska Native (0.7%), Asian or Pacific Islander (11.6%), non-Hispanic Black (12.4%), Hispanic (17.0%), and non-Hispanic White (58.3%). The study reported a notable rise in incidence rates, particularly after 2016. The highest age-standardized incidence rates were observed among non-Hispanic Black women aged 20–29 and 30–39. Furthermore, non-Hispanic Black women had the highest rates of advanced-stage disease, while Hispanic women experienced the largest increase in incidence rates compared to other racial and ethnic groups.^2^ The issue of early-onset BC and racial disparities in incidence and mortality is concerning for scientists, health care providers, policy makers, and public health officials. Preventing cancers and reducing cancer health disparities are national priorities in promoting better health for all. With the increasing trends in BC incidence, this public health issue is particularly pressing for young racial and ethnic minority women, who bear a disproportionate burden from breast cancer, and experience barriers to care due to social determinants of health (SDOH). Black and Hispanic women are particularly vulnerable due to younger ages at diagnosis, more aggressive subtypes, and higher risk of death from BC.^1,2,22^

Similar to our case, Chen et al. reported a striking case in the *New England Journal of Medicine* of a 14-year-old adolescent with triple-negative breast cancer (TNBC).^9^ The patient experienced metastasis 1 year and 4 months after surgery, a radical mastectomy of the breast mass. She was found to have pathogenic variants in both *BRCA1* and *TP53* genes, highlighting the biologically aggressive and complex medical nature of BC in adolescents and young adults.^9^ In our study, a 19 year-old is at a critical life stage, transitioning into young adulthood, which presents unique psychosocial challenges, including pursuing a college education, attaining financial security and consideration of supporting themselves. There are also significant mental health challenges experienced by this population, including fear of recurrence when diagnosed at a young age.^26,27^ There is an urgent need to gather more data on early-onset breast cancer cases and to investigate the interplay between biological, psychological, social and environmental factors, as well as to address disparities in outcomes for young women, particularly those from historically underserved communities.

*BRCA1* and *BRCA2* are the most common pathogenic variants associated with hereditary breast and ovarian cancer syndrome.^12^ However, our case highlights the importance of completing multi-gene panel testing to identify non-*BRCA* genes that are associated with hereditary BC to facilitate precision medicine and risk-reduction strategies.^14^ Our case had a *PTEN* (Phosphatase and Tensin Homologue) pathogenic variant. *PTEN* pathogenic variants are rare, occurring in approximately 1 in 200,000 individuals, and are linked to *PTEN* Hamartoma Tumor Syndrome (PHTS), which includes Cowden syndrome, Bannayan-Riley-Ruvalcaba syndrome, and Proteus/Proteus-like syndrome.^20^ This case highlight the importance of multigene panel testing for hereditary breast and ovarian cancer (HBOC) syndrome and its implications for at-risk family members, who can benefit from cascade testing to inform decision-making and take action to reduce their risk of developing cancer.^28^

## Implications for Practice

Clinicians, particularly nurses who are on the front lines of healthcare, play a major role in caring for the newly diagnosed young adult women with BC and in addressing the long-term effects of the disease among YBCS. Being diagnosed with BC at age 19 can have profound physical, emotional, social, and practical consequences, as young adults are at a critical life-stage, pursing a college education, and entering into relationships. Thus, nurses are uniquely positioned to care for young women with BC through a holistic, person-centered, caring science approach, that addresses physical, psychological, emotional, environmental, and social needs.

Nursing by its essence is a caring, healing, and holistic discipline. Nurses are pioneers in *caring science*, which focuses on wholeness, consciousness, and considers what matters most to those being cared for.^29–32^ Dr. Jean Watson’s Human Caring Theory provides a philosophy that is grounded in caring that can be useful in healthcare.^30,33^ Nurses can apply this theory to care for young adult women with BC holistically. It is essential to screen for depression and anxiety and to connect recently diagnosed patients and YBCS to mental health professionals, and provide resources to address the psychological and social aspects of a cancer diagnosis. One critical aspect of our case is that the patient stated that she never connected with another young cancer survivor of similar age during her cancer journey. For other newly diagnosed young women, peer support is an important form of psychosocial support that has been shown to reduce fear of recurrence and improve overall quality of life (QoL).^27^ By bridging gaps in emotional support and connecting patients to these resources, nurses can help to empower young survivors, fostering resilience and improving health outcomes, as nurses are well-positioned to connect young women to patient advocacy groups and peer support organizations^33^ This comprehensive approach ensures these young women not only survive but thrive, despite the challenges of a BC diagnosis.

## Patient Advocacy and Support Organizations

Numerous patient advocacy organizations and support groups are available for young breast cancer patients and YBCS, which providers can use as referral resources. Resources are available for patients living in underserved communities and to provide them access to quality healthcare and patient navigation services. Young breast cancer patients and survivors often face unique challenges that require tailored support and resources for their age group. Numerous patient advocacy organizations and support groups are dedicated to addressing these needs of young women. These organizations provide essential services, including education, peer support, financial assistance, and navigation through the cancer care continuum. Table 1 presents patient advocacy organizations.

## Health Policy Implications

Nurses play a vital role in shaping health policies and advocating for the populations they serve to improve health outcomes. Research indicates that younger women often face diagnostic delays due to lack of routine breast cancer screening before age 40.^11^ In response to growing evidence, the U.S. Preventive Services Task Force (USPSTF) updated its screening recommendations in 2024, advising that all women begin biennial screening mammograms at age 40, rather than age 50 years old.^34^

While mammograms save lives through early detection, and regular screening significantly lowers the risk of dying from BC;^35,36^ screening mammograms are not the standard of care for women under 40 years. This highlights the importance of health policies like the *Find It Early Act*, which holds particular promise for younger women, especially those with dense breast tissue or a higher risk of breast cancer. The policy emphasizes early detection, critical for younger women who often develop more aggressive forms of breast cancer. By ensuring access to advanced diagnostic tools and screening technologies, the *Find It Early Act* has the potential to improve breast health outcomes, facilitate timely access to screening and treatment, and reduce the overall burden of BC for vulnerable under-represented populations.

The National Cancer Institute’s *National Cancer Plan*^37^ also prioritizes cancer prevention as one of its eight goals, stressing the importance of reducing risk factors and staying up to date with cancer screenings. Nurses are uniquely positioned to advocate for such policies and educate younger women on prevention and early detection strategies. The limited screening recommendations for women under 40 further underscore the need for a comprehensive breast health awareness campaign targeting very young women. These efforts can empower them to understand their bodies, recognize potential warning signs, and promote self-advocacy, ultimately improving early detection and health outcomes for this underserved group.

## Prevention and Early Detection

For prevention, there is a need to increase conversations surrounding the incidence of breast cancer in young women, the associated risk factors, and strategies to reduce cancer risk starting at an early age. An objective assessment of risk using breast cancer risk assessment tools can be beneficial, as a woman’s risk is complex and based on biological, genetic, lifestyle, and environmental factors.^38^ Tools like iPrevent provide risk management options tailored to an individual’s risk category.^38^ For young adult women under 40 experiencing breast symptoms, cancer may not be on their radar. However, as demonstrated in our case report, it’s critical to be seen by a healthcare provider as soon as symptoms appear. Below we present the most common symptoms.

COMMON WARNING SIGNS AND SYMPTOMS:

- **Lump:** The most common symptom of breast cancer is a new lump in the breast. It is important to learn the normal feel of your breasts and be aware of any changes. Tumors are often firm and may feel different from normal breast tissue.
- **A painless, hard mass** with irregular edges is more likely to be cancer, although some cancers can be soft, round, tender, or painful. Thickening that feels different from surrounding tissue should be evaluated by a healthcare provider.
- **Nipple Discharge**: (other than breast milk) abnormal or bloody fluid from breast.
- **Skin Changes:** Dimpling that sometimes looks like an orange peel, swelling, thickening, dry, flaky, puckering, or redness. Changes to skin texture.
- **Swelling** of all or part of the breast, even in the absence of a lump.
- **Nipple retraction:** A change where the nipple turns inward.
- **Change in breast size, shape, or color**: Unexplained shrinkage of one breast or noticeable change in size or color. Thickening, pitting, flaking, irritation of the skin.
- **Lymph Node Changes**: Under the arm or near the collarbone. This can sometimes indicate the spread of breast cancer, even before the original tumor in the breast is large enough to be felt.
- **Breast or Nipple Pain:** Persistent or localized breast pain in one area of the breast or nipple or heaviness or radiating pain.

## Conclusion

This article highlights the increasing incidence of breast cancer (BC) among young women, emphasizing the need for more comprehensive research on early-onset BC. It calls for a deeper exploration of the complex interaction between biological, psychological, social, and environmental risk factors, as well as an examination of the racial and ethnic differences that influence BC outcomes. There is a critical need to develop interventions that are tailored to the cultural and age-specific needs of racial and ethnic minority women to promote cancer risk-reduction and to improve health outcomes. The case report highlights the importance of multigene panel testing in identifying genetic risks, particularly for young women, who are often diagnosed with more aggressive forms of cancer. Furthermore, it emphasizes the importance of a holistic approach to care, grounded in caring science, which prioritizes the well-being of the whole person. Nurses, in collaboration with other healthcare providers, are in a unique position to positively impact the lives of young breast cancer patients, survivors, and those at high-risk for developing the disease. By advocating for early detection, tailored age-appropriate interventions, and comprehensive support, nurses can significantly improve health outcomes and empower young women to live longer, healthier lives. Future research should investigate the complex interaction between biological, psychological, social, and environmental factors in order to reduce cancer health disparities and improve health for all.

## Figures and Tables

**Figure 1.**
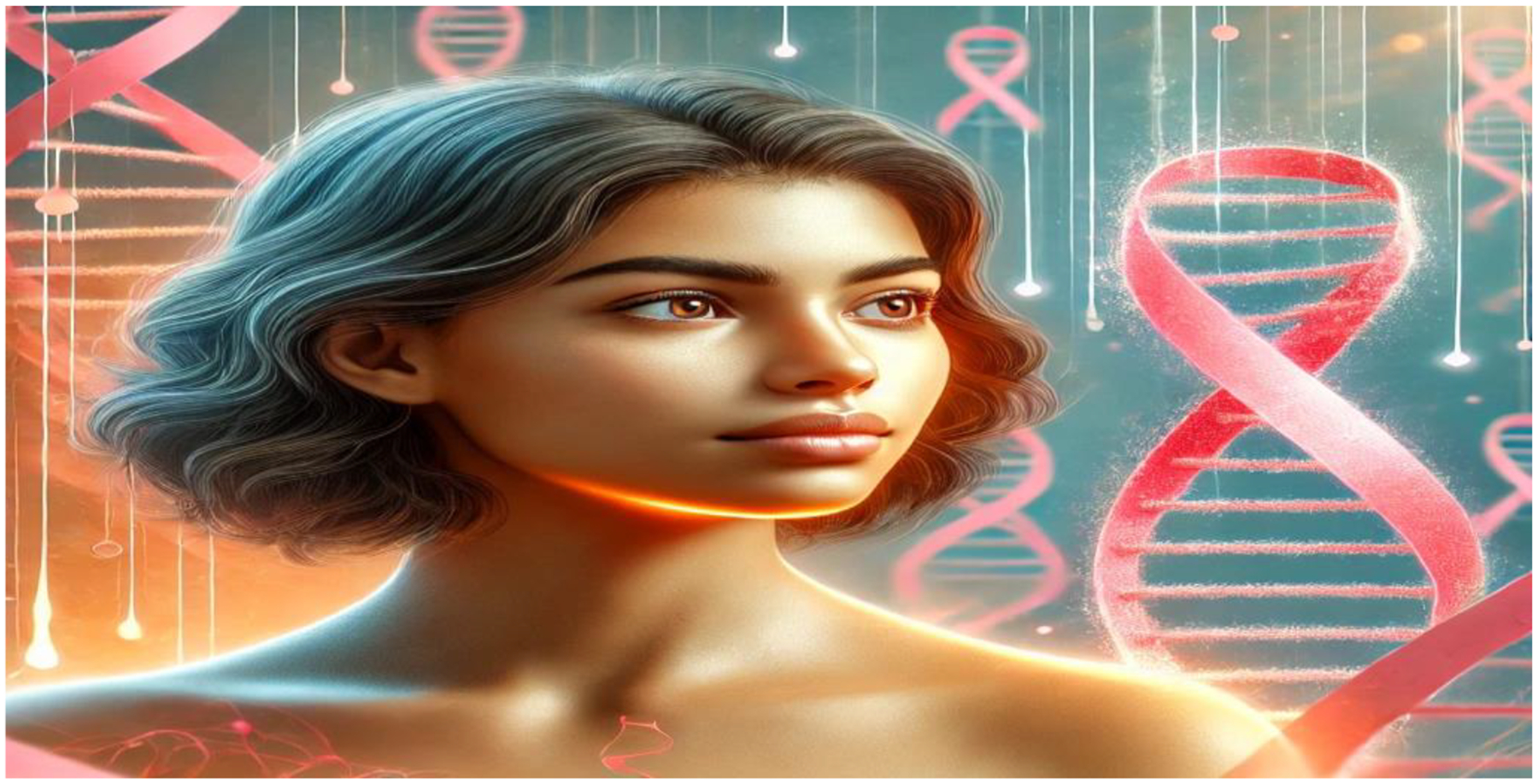
Artistic depiction of a young adult woman with breast cancer **Source Credit:** OpenAI. (2024). [Digital illustration]. DALL·E. https://openai.com/dalle

**Figure 2.**
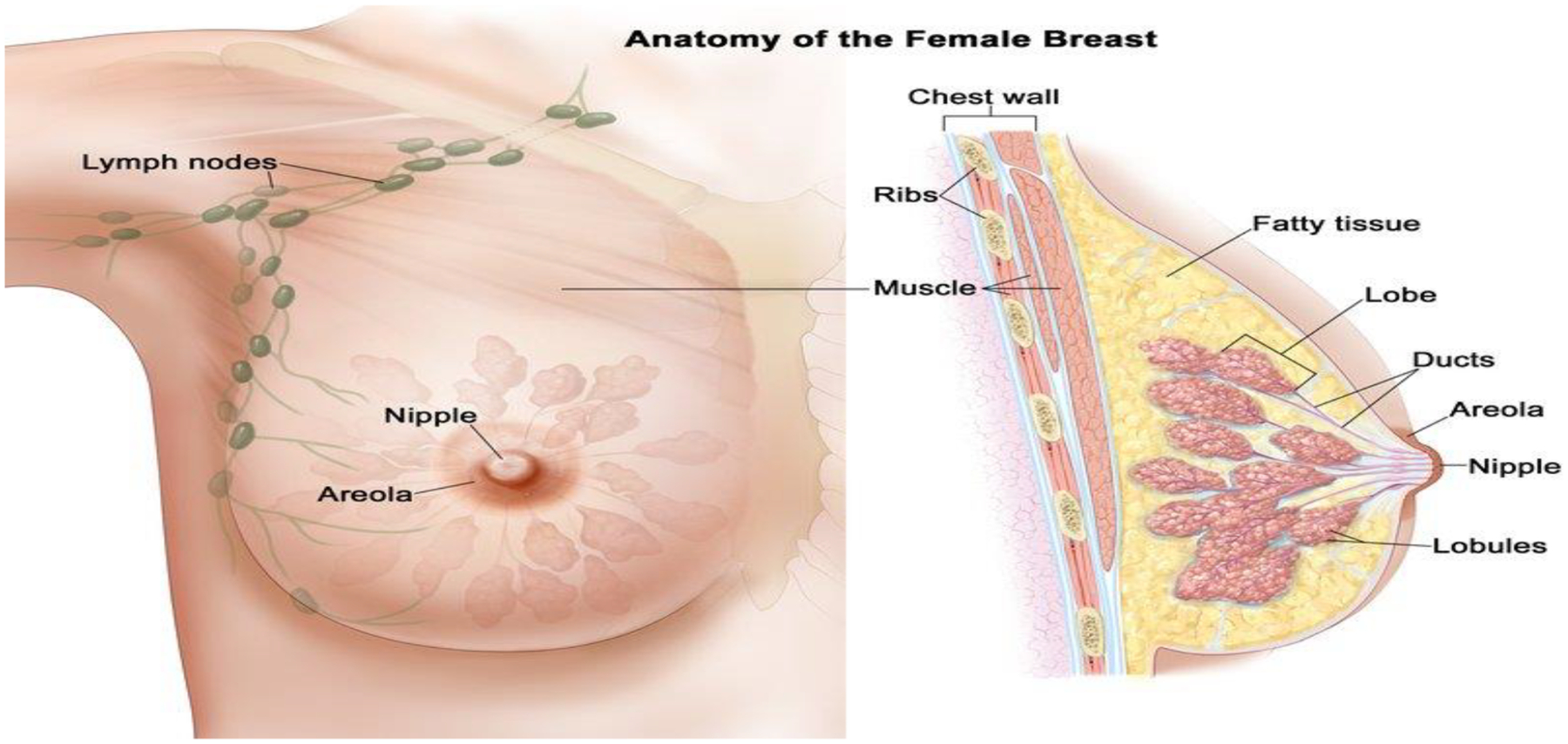
Female Breast Anatomy National Cancer Institute (NCI) Visuals Online. Drawing of female breast anatomy. Retrieved from: https://visualsonline.cancer.gov/details.cfm?imageid=7127

**Figure 3.**
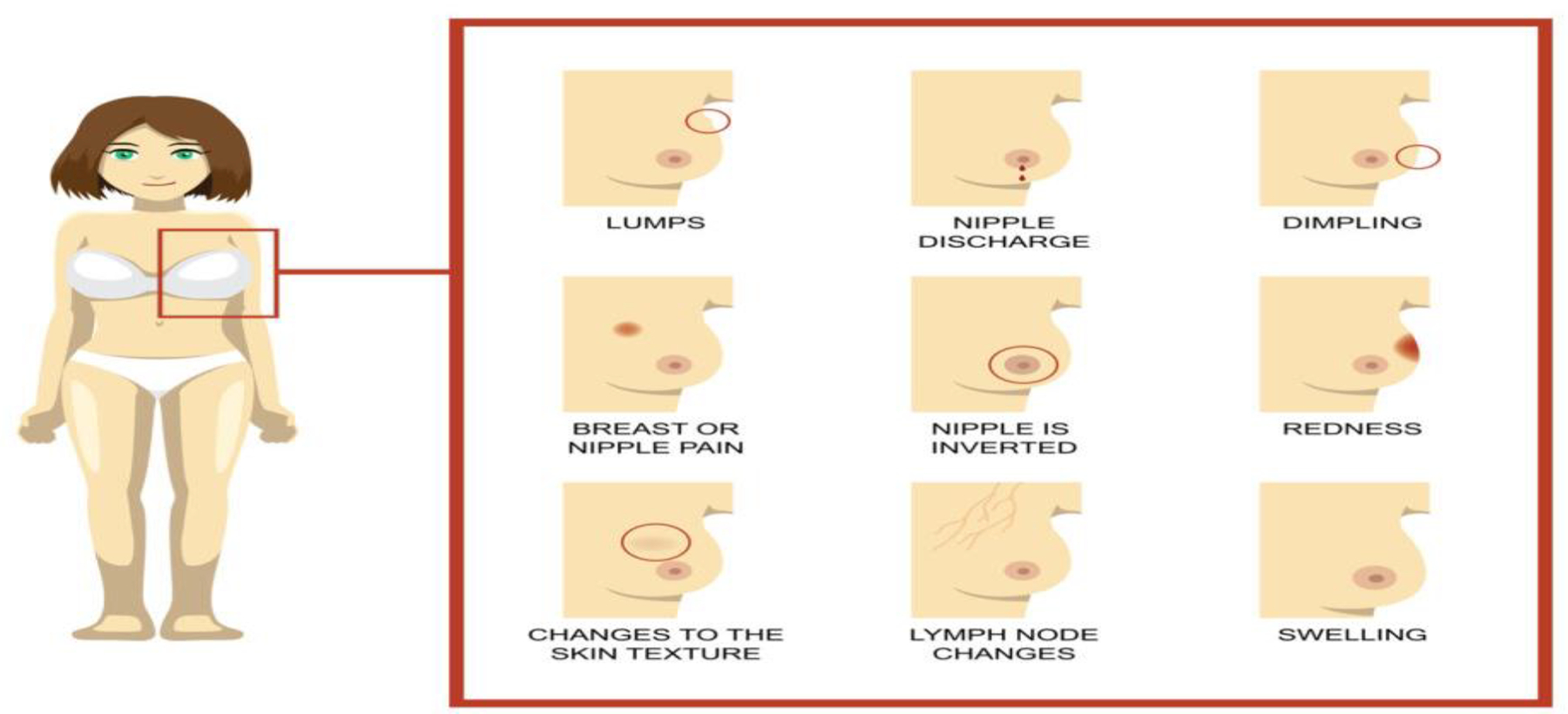
Breast cancer common signs and symptoms Oncology Nurse Advisor: Retrieved from: https://www.oncologynurseadvisor.com/features/breast-cancer-symptoms/ **Note.** Discuss breast cancer risk, concerns, symptoms, when to begin screening and the timing of your mammogram with a health care provider.

**Table 1:** Breast Cancer Advocacy and Support Organizations for Young Women

Organization	Focus Area	Website/Contact
FAU/NCHA Community Health Center	Provides healthcare and support to diverse underserved populations and communities who otherwise could not afford or access quality care. A patient navigator is available to assist with breast health needs across the cancer care continuum.	https://www.faunursing.org/ncha/
**ACS CARES^™^ (Community Access to Resources, Education, and Support)**	A free mobile app from the American Cancer Society designed to support individuals with cancer, their families, and caregivers. It offers tools and resources to manage care, access information, and find support throughout the cancer journey.	https://www.cancer.org/support-programs-and-services/acs-cares.html
Tigerlily Foundation	Empowers women aged 15–45 through education, advocacy, and support for breast health and awareness.	www.tigerlilyfoundation.org
Young Survival Coalition	Supports young women under 40 diagnosed with breast cancer through resources, programs, and events.	www.youngsurvival.org
Living Beyond Breast Cancer	Provides information, support, and community for all people impacted by breast cancer.	www.lbbc.org
Triple Negative Breast Cancer Foundation	Focuses on raising awareness, providing resources, and funding research for TNBC.	www.tnbcfoundation.org
Florida Breast Cancer Foundation	Advocates for breast cancer education, research, and prevention in Florida.	www.floridabreastcancer.org
The Women’s Breast & Heart Initiative	Promotes breast and heart health through education, access to screenings, and community outreach.	www.flbreasthealth.com
The Pink Queen Foundation	Provides financial assistance and emotional support to underserved individuals battling breast cancer.	www.thepinkqueenfoundation.org
Promise Fund	Supports women with breast and cervical cancer through financial aid, care navigation, and outreach.	www.promisefundofflorida.org
Breast cancer in Young Women	Focuses on educating and empowering young women about breast cancer risks and treatment.	www.breastcancerinyoungwomen.org
Keep a Breast Foundation	Promotes breast cancer prevention through education, awareness, and self-check resources	https://www.keep-a-breast.org/
PROACT Program	Provides support and resources for young women with breast cancer.	https://proactprogram.org/
